# 2,6-Dide­oxy-2,6-imino-l-*glycero*-d-*ido*-heptitol

**DOI:** 10.1107/S1600536809025045

**Published:** 2009-07-04

**Authors:** Sarah F. Jenkinson, K. Victoria Booth, Scott Newberry, George W. J. Fleet, Ken Izumori, Kenji Morimoto, Robert J. Nash, Laurence Jones, David J. Watkin, Amber L. Thompson

**Affiliations:** a Department of Organic Chemistry, Chemistry Research Laboratory, Department of Chemistry, University of Oxford, Oxford OX1 3TA, England; b Rare Sugar Research Centre, Kagawa University, 2393 Miki-cho, Kita-gun, Kagawa 761-0795, Japan; c Summit PLC, Plas Gogerddan, Aberystwyth, Ceredigion SY23 3EB, Wales; d Department of Chemical Crystallography, Chemistry Research Laboratory, Department of Chemistry, University of Oxford, Oxford OX1 3TA, England

## Abstract

The title mol­ecule, C_7_H_15_NO_5_, the major product from selective enzymatic oxidation followed by hydrogeno­lysis of the corresponding azido­heptitol, was found by X-ray crystallography to exisit in a chair conformation with three axial hydroxyl groups. One of the hydroxymethyl groups is disordered over two sets of sites in a 0.590 (3):0.410 (3) ratio.   In the crystal, O—H⋯O, O—H⋯(O,O), O—H⋯N and N—H⋯O hydrogen bonding occurs.

## Related literature

For the synthesis of homonojirimycin derivatives, see: Compain *et al.* (2009 ▶); Asano *et al.* (2000 ▶); Watson *et al.* (2001 ▶); Ikeda *et al*. (2000 ▶); Asano *et al.* (1998 ▶); Kite *et al.* (1988 ▶); Dondoni & Nuzzi (2006 ▶). For the biological applications of homonojirimycin derivatives, see: Compain *et al.* (2006 ▶). For related literature on Izumoring technology, see: Izumori *et al.* (2002 ▶, 2006 ▶); Yoshihara *et al.* (2008 ▶); Rao *et al.* (2008 ▶); Jones *et al.* (2008 ▶). For related crystallography literature, see: Görbitz (1999 ▶).
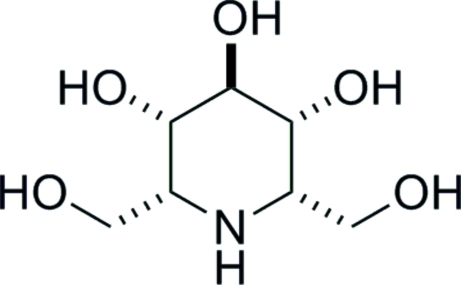


## Experimental

### 

#### Crystal data

C_7_H_15_NO_5_
*M* *_r_* = 193.20
Monoclinic, 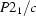

*a* = 10.2907 (3) Å
*b* = 7.6035 (3) Å
*c* = 11.0057 (3) Å
β = 91.8668 (16)°
*V* = 860.69 (5) Å^3^
*Z* = 4
Mo *K*α radiation
μ = 0.13 mm^−1^
*T* = 150 K
0.50 × 0.50 × 0.20 mm

#### Data collection

Area diffractometer
Absorption correction: multi-scan (*DENZO*/*SCALEPACK*; Otwinowski & Minor, 1997 ▶) *T*_min_ = 0.81, *T*_max_ = 0.98
7892 measured reflections
1944 independent reflections
1609 reflections with *I* > 2σ(*I*)
*R*_int_ = 0.030

#### Refinement

*R*[*F* ^2^ > 2σ(*F* ^2^)] = 0.039
*wR*(*F* ^2^) = 0.097
*S* = 0.99
1944 reflections
137 parameters
H-atom parameters constrained
Δρ_max_ = 0.33 e Å^−3^
Δρ_min_ = −0.32 e Å^−3^

### 

Data collection: *COLLECT* (Nonius, 2001 ▶); cell refinement: *DENZO*/*SCALEPACK* (Otwinowski & Minor, 1997 ▶); data reduction: *DENZO*/*SCALEPACK*; program(s) used to solve structure: *SIR92* (Altomare *et al.*, 1994 ▶); program(s) used to refine structure: *CRYSTALS* (Betteridge *et al.*, 2003 ▶); molecular graphics: *CAMERON* (Watkin *et al.*, 1996 ▶); software used to prepare material for publication: *CRYSTALS*.

## Supplementary Material

Crystal structure: contains datablocks I, global. DOI: 10.1107/S1600536809025045/lh2854sup1.cif

Structure factors: contains datablocks I. DOI: 10.1107/S1600536809025045/lh2854Isup2.hkl

Additional supplementary materials:  crystallographic information; 3D view; checkCIF report

## Figures and Tables

**Table 1:** Hydrogen-bond geometry (Å, °)

*D*—H⋯*A*	*D*—H	H⋯*A*	*D*⋯*A*	*D*—H⋯*A*
O14—H141⋯O16^i^	0.80	1.89	2.684 (3)	170
O16—H161⋯N1^ii^	0.84	1.96	2.793 (3)	171
N1—H11⋯O12^iii^	0.84	2.17	2.996 (3)	165
O13—H131⋯O8^iv^	0.79	1.97	2.739 (3)	165
O13—H131⋯O11^iv^	0.79	2.08	2.824 (3)	158
O12—H121⋯O14^v^	0.85	2.39	3.052 (3)	136
O8—H81⋯O13^vi^	0.83	2.00	2.805 (3)	164
O11—H111⋯O13^vi^	0.82	2.02	2.843 (3)	173

Symmetry codes: (i) 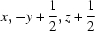; (ii) 
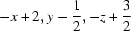
; (iii) 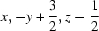; (iv) 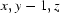; (v) 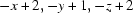; (vi) 
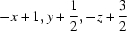
.

**Fig. 1.**
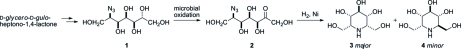
Synthetic Scheme.

**Fig. 2.**
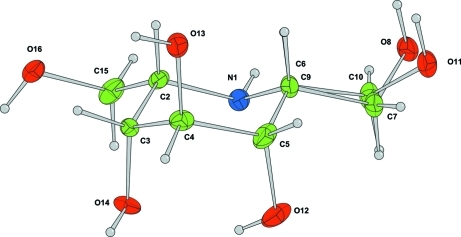
The title compound with displacement ellipsoids drawn at the 50% probability level. H atoms are shown as spheres of arbitary radius.

**Fig. 3.**
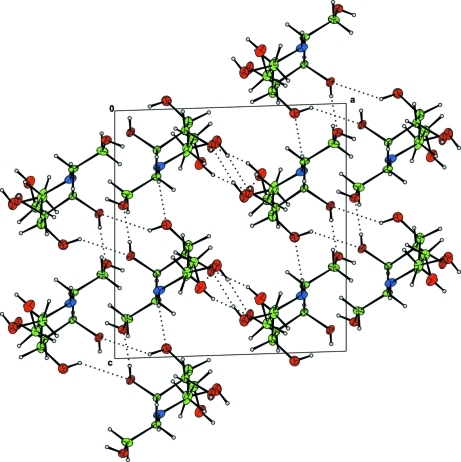
Packing diagram for the title compound. The compound exists as an extensively hydrogen bonded network (dotted lines).

## supplementary crystallographic information

### Comment

The methodology developed by Izumori (2002, 2006) for the interconversion of tetroses, pentoses and hexoses by enzymatic oxidation, inversion at C3 with a single epimerase, and reduction to the aldose has been seen to be generally applicable for the 1-deoxy ketohexoses (Yoshihara *et al.*, 2008) and branched sugars (Rao *et al.*, 2008; Jones *et al.*, 2008). This methodology has now also been applied to azido heptitols and thus to the synthesis of 2,6-dideoxy-2,6-iminoheptitols (homonojirimycins); these seven carbon imino sugars (Compain *et al.*, 2009; Asano *et al.*, 2000; Watson *et al.*, 2001), are a family of glycosidase inhibitors. A number of homonojrimycins have been isolated as natural products from medicinal plants (Ikeda *et al.*, 2000; Asano *et al.*, 1998; Kite *et al.*, 1988). Other piperidines with all the ring hydroxyl groups axial have been shown to be very powerful glycosidase inhibitors (Compain *et al.*, 2006).

The azido heptitol 1 was synthesized from readily available D-*glycero*-D-*gulo*-heptono-1,4-lactone and underwent selective enzymatic oxidation to the ketose 2 followed by hydrogenation with closure on either face of the ketone to generate the imino sugars 3 and 4 (Fig. 1). The major product was found to be the symmetrical homonorjirimycin 3 and its structure was confirmed by X-ray crystallography.

The X-ray structure shows that the compound adopts a chair conformation with 3 axial hydroxyl substituents (Fig. 2). There is significant disorder in the structure with one of the equatorial hydroxymethyl groups occupying two possible sites each of which is able to form a hydrogen bond.The crystal exists as an extensively hydrogen bonded lattice with each molecule acting as a donor and an acceptor for 8 hydrogen bonds (Fig. 3).

### Experimental

The title compound was recrystallized from mixture of 95% ethanol and 5% water layered with acetone: m.p. 442–445 K (free base); [α]_D_^25^ 0.0 (*c*, 1.27 in MeOH) (HCl salt). All other data was consistent with the literature data for the HCl salt (Dondoni & Nuzzi, 2006).

### Refinement

The relatively large ratio of minimum to maximum corrections applied in the multiscan process (1:1.21) reflect changes in the illuminated volume of the crystal. Changes in illuminated volume were kept to a minimum, and were taken into account (Görbitz, 1999) by the multi-scan inter-frame scaling (*DENZO*/*SCALEPACK*, Otwinowski & Minor, 1997).

The H atoms were all located in a difference map, but those attached to carbon atoms were repositioned geometrically. The H atoms were initially refined with soft restraints on the bond lengths and angles to regularize their geometry (C—H in the range 0.93–0.98, N—H in the range 0.86–0.89 N—H to 0.86 O—H = 0.82 Å) and *U*_iso_(H) (in the range 1.2–1.5 times *U*_eq_ of the parent atom), after which the positions were refined with riding constraints.

### Crystal data

**Table:**

C_7_H_15_NO_5_	*F*(000) = 416
*M**_r_* = 193.20	*D*_x_ = 1.491 Mg m^−^^3^
Monoclinic, *P*2_1_/*c*	Melting point = 442–445 K
Hall symbol: -P 2ybc	Mo *K*α radiation, λ = 0.71073 Å
*a* = 10.2907 (3) Å	Cell parameters from 1957 reflections
*b* = 7.6035 (3) Å	θ = 5–27°
*c* = 11.0057 (3) Å	µ = 0.13 mm^−^^1^
β = 91.8668 (16)°	*T* = 150 K
*V* = 860.69 (5) Å^3^	Block, colourless
*Z* = 4	0.50 × 0.50 × 0.20 mm

### Data collection

**Table:**

Area diffractometer	1609 reflections with *I* > 2σ(*I*)
graphite	*R*_int_ = 0.030
ω scans	θ_max_ = 27.5°, θ_min_ = 5.2°
Absorption correction: multi-scan (*DENZO*/*SCALEPACK*; Otwinowski & Minor, 1997)	*h* = −13→13
*T*_min_ = 0.81, *T*_max_ = 0.98	*k* = −9→9
7892 measured reflections	*l* = −14→14
1944 independent reflections	

### Refinement

**Table:**

Refinement on *F*^2^	Primary atom site location: structure-invariant direct methods
Least-squares matrix: full	Hydrogen site location: inferred from neighbouring sites
*R*[*F*^2^ > 2σ(*F*^2^)] = 0.039	H-atom parameters constrained
*wR*(*F*^2^) = 0.097	*w* = 1/[σ^2^(*F*^2^) + (0.04*P*)^2^ + 0.51*P*], where *P* = [max(*F*_o_^2^,0) + 2*F*_c_^2^]/3
*S* = 1.00	(Δ/σ)_max_ = 0.0003
1944 reflections	Δρ_max_ = 0.33 e Å^−^^3^
137 parameters	Δρ_min_ = −0.32 e Å^−^^3^
0 restraints	

### Fractional atomic coordinates and isotropic or equivalent isotropic displacement parameters (Å^2^)

**Table:**

	*x*	*y*	*z*	*U*_iso_*/*U*_eq_	Occ. (<1)
N1	0.80811 (11)	0.66935 (14)	0.76944 (10)	0.0174	
C2	0.82099 (13)	0.48495 (17)	0.72876 (11)	0.0140	
C3	0.81825 (12)	0.36103 (16)	0.83746 (11)	0.0123	
C4	0.69637 (12)	0.38977 (18)	0.91183 (12)	0.0165	
C5	0.68068 (13)	0.5840 (2)	0.94446 (13)	0.0224	
C6	0.68515 (14)	0.69847 (18)	0.83143 (14)	0.0233	0.590 (3)
C7	0.6593 (4)	0.8833 (5)	0.8777 (4)	0.0176	0.590 (3)
O8	0.63068 (17)	0.9958 (2)	0.77658 (16)	0.0216	0.590 (3)
C9	0.68515 (14)	0.69847 (18)	0.83143 (14)	0.0233	0.410 (3)
C10	0.6878 (5)	0.9060 (7)	0.8365 (5)	0.0181	0.410 (3)
O11	0.5595 (2)	0.9664 (3)	0.8625 (2)	0.0216	0.410 (3)
O12	0.77676 (10)	0.63650 (15)	1.03344 (9)	0.0255	
O13	0.58223 (9)	0.33556 (13)	0.84432 (9)	0.0203	
O14	0.93148 (8)	0.39490 (12)	0.91177 (8)	0.0165	
C15	0.94645 (14)	0.46860 (18)	0.66037 (12)	0.0197	
O16	0.96317 (10)	0.29326 (13)	0.61694 (8)	0.0198	
H21	0.7458	0.4528	0.6738	0.0157*	
H31	0.8191	0.2378	0.8090	0.0130*	
H41	0.7061	0.3208	0.9884	0.0187*	
H61	0.6119	0.6629	0.7752	0.0259*	0.590 (3)
H72	0.7376	0.9276	0.9206	0.0197*	0.590 (3)
H71	0.5868	0.8832	0.9358	0.0206*	0.590 (3)
H91	0.6128	0.6622	0.7753	0.0259*	0.410 (3)
H101	0.7493	0.9437	0.9007	0.0210*	0.410 (3)
H102	0.7164	0.9563	0.7589	0.0216*	0.410 (3)
H152	1.0213	0.4990	0.7155	0.0218*	
H151	0.9433	0.5524	0.5897	0.0228*	
H141	0.9354	0.3293	0.9687	0.0234*	
H161	1.0333	0.2501	0.6444	0.0297*	
H51	0.5938	0.5992	0.9815	0.0254*	
H11	0.8100	0.7367	0.7086	0.0211*	
H131	0.5842	0.2333	0.8312	0.0306*	
H121	0.8487	0.5930	1.0131	0.0367*	
H81	0.5752	0.9542	0.7287	0.0322*	0.590 (3)
H111	0.5126	0.9323	0.8051	0.0290*	0.410 (3)

### Atomic displacement parameters (Å^2^)

**Table:**

	*U*^11^	*U*^22^	*U*^33^	*U*^12^	*U*^13^	*U*^23^
N1	0.0274 (6)	0.0091 (5)	0.0154 (5)	0.0007 (4)	−0.0047 (5)	0.0028 (4)
C2	0.0195 (6)	0.0112 (6)	0.0112 (6)	−0.0011 (5)	−0.0025 (5)	−0.0008 (5)
C3	0.0128 (6)	0.0108 (6)	0.0131 (6)	0.0006 (5)	−0.0020 (5)	0.0003 (5)
C4	0.0124 (6)	0.0209 (7)	0.0162 (6)	−0.0015 (5)	−0.0006 (5)	−0.0015 (5)
C5	0.0140 (6)	0.0270 (8)	0.0261 (7)	−0.0001 (5)	0.0015 (5)	−0.0142 (6)
C6	0.0182 (7)	0.0147 (7)	0.0360 (8)	0.0060 (5)	−0.0121 (6)	−0.0108 (6)
C7	0.0178 (18)	0.0121 (15)	0.0228 (19)	0.0025 (12)	−0.0007 (13)	−0.0022 (14)
O8	0.0237 (10)	0.0108 (8)	0.0296 (10)	0.0017 (7)	−0.0075 (8)	0.0001 (7)
C9	0.0182 (7)	0.0147 (7)	0.0360 (8)	0.0060 (5)	−0.0121 (6)	−0.0108 (6)
C10	0.016 (2)	0.011 (2)	0.027 (3)	0.0000 (17)	0.0016 (19)	−0.003 (2)
O11	0.0181 (13)	0.0185 (13)	0.0281 (14)	0.0063 (9)	−0.0002 (10)	−0.0055 (10)
O12	0.0200 (5)	0.0358 (6)	0.0207 (5)	−0.0033 (4)	0.0012 (4)	−0.0153 (4)
O13	0.0139 (5)	0.0153 (5)	0.0313 (5)	−0.0006 (4)	−0.0040 (4)	−0.0058 (4)
O14	0.0143 (5)	0.0191 (5)	0.0158 (4)	−0.0021 (4)	−0.0047 (3)	0.0067 (4)
C15	0.0268 (7)	0.0179 (7)	0.0147 (6)	−0.0055 (5)	0.0042 (5)	−0.0021 (5)
O16	0.0218 (5)	0.0215 (5)	0.0159 (4)	0.0025 (4)	−0.0009 (4)	−0.0073 (4)

### Geometric parameters (Å, °)

**Table:**

N1—C2	1.4791 (16)	C7—O8	1.427 (4)
N1—C6	1.4738 (19)	C7—H72	0.981
N1—H11	0.844	C7—H71	0.998
C2—C3	1.5238 (17)	O8—H81	0.827
C2—C15	1.5206 (19)	C9—C10	1.579 (6)
C2—H21	0.997	C9—H91	0.991
C3—C4	1.5355 (17)	C10—O11	1.435 (6)
C3—O14	1.4251 (14)	C10—H101	0.976
C3—H31	0.988	C10—H102	0.989
C4—C5	1.5295 (19)	O11—H111	0.824
C4—O13	1.4300 (15)	O12—H121	0.847
C4—H41	0.995	O13—H131	0.791
C5—C6	1.520 (2)	O14—H141	0.801
C5—O12	1.4258 (16)	C15—O16	1.4286 (16)
C5—H51	1.002	C15—H152	0.992
C6—C7	1.521 (4)	C15—H151	1.005
C6—H61	0.997	O16—H161	0.840
			
C2—N1—C6	111.67 (10)	C7—C6—H61	108.8
C2—N1—H11	109.3	C6—C7—O8	109.1 (3)
C6—N1—H11	108.6	C6—C7—H72	109.2
N1—C2—C3	110.14 (10)	O8—C7—H72	108.4
N1—C2—C15	108.25 (10)	C6—C7—H71	110.9
C3—C2—C15	112.07 (11)	O8—C7—H71	111.1
N1—C2—H21	109.9	H72—C7—H71	108.1
C3—C2—H21	106.9	C7—O8—H81	112.8
C15—C2—H21	109.6	C5—C9—N1	110.01 (11)
C2—C3—C4	111.52 (10)	C5—C9—C10	123.0 (2)
C2—C3—O14	107.64 (10)	N1—C9—C10	98.8 (2)
C4—C3—O14	109.59 (10)	C5—C9—H91	108.0
C2—C3—H31	109.7	N1—C9—H91	108.0
C4—C3—H31	108.6	C10—C9—H91	108.2
O14—C3—H31	109.8	C9—C10—O11	108.2 (4)
C3—C4—C5	110.91 (11)	C9—C10—H101	109.2
C3—C4—O13	110.69 (10)	O11—C10—H101	110.0
C5—C4—O13	108.00 (11)	C9—C10—H102	111.2
C3—C4—H41	108.4	O11—C10—H102	110.4
C5—C4—H41	108.6	H101—C10—H102	107.9
O13—C4—H41	110.3	C10—O11—H111	105.5
C4—C5—C6	110.76 (11)	C5—O12—H121	107.5
C4—C5—O12	110.82 (12)	C4—O13—H131	110.7
C6—C5—O12	111.27 (12)	C3—O14—H141	110.9
C4—C5—H51	108.0	C2—C15—O16	110.82 (11)
C6—C5—H51	108.6	C2—C15—H152	109.5
O12—C5—H51	107.2	O16—C15—H152	108.8
C5—C6—N1	110.01 (11)	C2—C15—H151	108.9
C5—C6—C7	104.18 (18)	O16—C15—H151	109.5
N1—C6—C7	117.11 (18)	H152—C15—H151	109.3
C5—C6—H61	108.1	C15—O16—H161	110.8
N1—C6—H61	108.3		

### Hydrogen-bond geometry (Å, °)

**Table:**

*D*—H···*A*	*D*—H	H···*A*	*D*···*A*	*D*—H···*A*
O11—H71···C5	1.06	2.47	3.279 (3)	132
O8—H102···N1	0.96	2.38	3.084 (3)	130
O14—H141···O16^i^	0.80	1.89	2.684 (3)	170
O16—H161···N1^ii^	0.84	1.96	2.793 (3)	171
N1—H11···O12^iii^	0.84	2.17	2.996 (3)	165
O13—H131···O8^iv^	0.79	1.97	2.739 (3)	165
O13—H131···O11^iv^	0.79	2.08	2.824 (3)	158
O12—H121···O14	0.85	2.07	2.800 (3)	143
O12—H121···O14^v^	0.85	2.39	3.052 (3)	136
O8—H81···O13^vi^	0.83	2.00	2.805 (3)	164
O11—H111···O13^vi^	0.82	2.02	2.843 (3)	173

Symmetry codes: (i) *x*, −*y*+1/2, *z*+1/2; (ii) −*x*+2, *y*−1/2, −*z*+3/2; (iii) *x*, −*y*+3/2, *z*−1/2; (iv) *x*, *y*−1, *z*; (v) −*x*+2, −*y*+1, −*z*+2; (vi) −*x*+1, *y*+1/2, −*z*+3/2.
